# Antimicrobial Peptide Expression at the Ocular Surface and Their Therapeutic Use in the Treatment of Microbial Keratitis

**DOI:** 10.3389/fmicb.2022.857735

**Published:** 2022-06-02

**Authors:** Allison H. Shannon, Sara A. Adelman, Erin A. Hisey, Sanskruti S. Potnis, Vanessa Rozo, Madeline W. Yung, Jennifer Y. Li, Christopher J. Murphy, Sara M. Thomasy, Brian C. Leonard

**Affiliations:** ^1^Department of Surgical and Radiological Sciences, School of Veterinary Medicine, University of California, Davis, Davis, CA, United States; ^2^William R. Pritchard Veterinary Medical Teaching Hospital, School of Veterinary Medicine, University of California, Davis, Davis, CA, United States; ^3^Department of Ophthalmology & Vision Science, School of Medicine, University of California, Davis, Davis, CA, United States

**Keywords:** antimicrobial peptides, cathelicidin (LL37), defensin, infectious keratitis, microbial keratitis

## Abstract

Microbial keratitis is a common cause of ocular pain and visual impairment worldwide. The ocular surface has a relatively paucicellular microbial community, mostly found in the conjunctiva, while the cornea would be considered relatively sterile. However, in patients with microbial keratitis, the cornea can be infected with multiple pathogens including *Staphylococcus aureus*, *Pseudomonas aeruginosa*, and *Fusarium* sp. Treatment with topical antimicrobials serves as the standard of care for microbial keratitis, however, due to high rates of pathogen resistance to current antimicrobial medications, alternative therapeutic strategies must be developed. Multiple studies have characterized the expression and activity of antimicrobial peptides (AMPs), endogenous peptides with key antimicrobial and wound healing properties, on the ocular surface. Recent studies and clinical trials provide promise for the use of AMPs as therapeutic agents. This article reviews the repertoire of AMPs expressed at the ocular surface, how expression of these AMPs can be modulated, and the potential for harnessing the AMPs as potential therapeutics for patients with microbial keratitis.

## Introduction

Microbial keratitis, or an infection of the cornea, can result in devastating visual impairment or even permanent vision loss if an effective, targeted therapy is not instituted in a timely manner. Damage to the corneal epithelium opens a portal for the entry of pathogenic microbes. This damage is often associated with contact lens use but can also be caused by a multitude of ocular surface traumas (Austin et al., 2017). With an incidence of one million visits to medical providers annually in the United States and an estimated $175 million in health expenditures, infectious keratitis places an enormous burden on patients and the healthcare system alike (Ung et al., 2019).

Antimicrobial peptides (AMPs) are key effector molecules with broad spectrum antimicrobial activity against bacteria, fungi, and viruses (Leonard et al., 2012; Boparai and Sharma, 2020). In addition to their direct antimicrobial activity, AMPs have been shown to modulate a wide array of critical cell behaviors including chemotaxis, cytokine production, epithelial cell proliferation, promotion of cell migration, angiogenesis, apoptosis, wound healing, and they even modulate coat color in dogs (Leonard et al., 2012). AMPs are small peptides, ranging anywhere from 12 to 50 amino acids in length, composed of cationic residues including arginine and lysine which are thought to exert the majority of their antimicrobial activity (McDermott, 2009). Additionally, these peptides contain hydrophilic and hydrophobic regions. The amphipathic nature of these peptides allows them to exert their antipathogenic action where they interact and directly incorporate into microbial cell walls and membranes (McDermott, 2009; Leonard et al., 2012).

AMPs are a component of the innate immune system expressed by epithelial cells (Fulton et al., 1997; Bals et al., 1998; Haynes et al., 1999; O’Neil et al., 1999) and leukocytes (Rivas-Santiago et al., 2008). Most epithelial cells that reside at the interface between pathogens and the host (i.e., mucosal surfaces such as the ocular surface, gastrointestinal tract, respiratory tract). These sites are where AMPs are constitutively expressed and increased expression can be induced by the presence of pathogenic or inflammatory stimuli (Haynes et al., 1999; McDermott et al., 2003; Rivas-Santiago et al., 2008). AMP expression can be upregulated to augment the antimicrobial activity at a body surface.

Given the rise in multidrug resistance, alternative and adjunctive treatment strategies for microbial keratitis are needed and the use of topically applied exogenous (natural and synthetic) AMPs and/or upregulation of endogenous AMPs may prove to be a critical therapeutic intervention. This review highlights the specific functions of AMPs expressed by the ocular surface and discusses the potential for exogenous treatment or modulation of host AMP expression as a therapeutic intervention for microbial keratitis.

## Antimicrobial Peptides at the Ocular Surface

**Figure 1:**
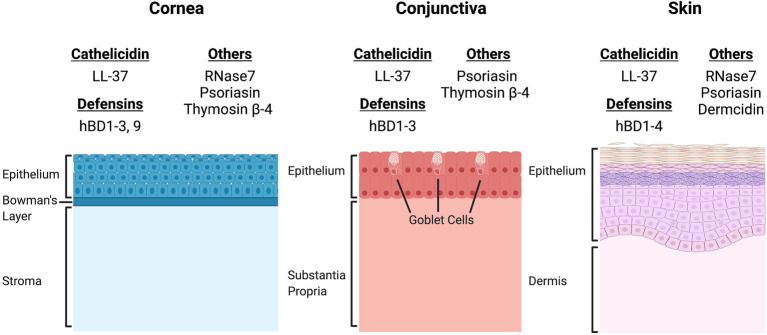
Comparison of the site-specific expression of antimicrobial peptides (AMPs) expressed by epithelial cells of the cornea, conjunctiva, and skin. There is significant overlap in the expression patterns of AMPs at both the ocular surface and the skin. Cathelicidin (LL-37), β-defensins 1, 2, and 3, and psoriasin are expressed by epithelial cells of the cornea, conjunctiva and skin (Herman and Herman, 2019). However, site specific expression has been documented for β-defensin 4 (hBD4, skin), hBD9 (cornea), RNase7 (cornea, skin), thymosin β-4 (cornea, conjunctiva) and dermcidin (skin). The differences in AMP expression patterns may directly influence the microbial niches of the cornea, conjunctiva, and skin.

There are many AMPs expressed throughout the body, however cathelicidin, defensins, histatin, thymosin, psoriasin, and ribonuclease-7 are the main AMPs that play key roles in the innate immune system of the ocular surface (Figure 1).

### Cathelicidins

Cathelicidins, named for their highly conserved cathelin domain at the N-terminus, were first discovered in bovine neutrophils and have since been documented in 133 other vertebrate species (Zanetti et al., 1990, 1995; Mookherjee et al., 2013). Many vertebrate species have multiple cathelicidins; but humans, mice, cats, and dogs are known to possess only one (Sang et al., 2007; Leonard et al., 2011; Mookherjee et al., 2013). The human cathelicidin antimicrobial peptide (hCAP18), encoded by the *CAMP* gene, is transcribed in a pro-form and is activated by specialized proteinases to become LL-37 (Sorensen et al., 2001; Bandurska et al., 2015). The LL-37 peptide is composed of 37 amino acids and is named for the two leucine residues found in the N-terminus of the mature peptide (Bandurska et al., 2015). Since its discovery in neutrophilic granules, LL-37 expression has been identified in various epithelial tissues including eye, skin, lung, mouth, tongue, esophagus, vagina, cervix, and sweat glands (Wang, 2014). At the ocular surface, LL-37 has both constitutive and inducible expression by conjunctival and corneal epithelial cells (McIntosh et al., 2005). The importance of their role in the innate immunity of the ocular surface was demonstrated through a study by Huang et al. (2007b), in which cathelicidin-deficient mice had increased susceptibility to corneal infection by *Pseudomonas aeruginosa*, delayed clearance of *P. aeruginosa*, and increased corneal infiltrating neutrophils when compared to wild type animals.

### Defensins

The defensins are encoded by multiple genes mainly clustered on human chromosome 8 (Jia et al., 2001, p. 22–23). The primary structure of defensin peptides differs from cathelicidins in that they range from 29 to 45 amino acids and contain six cysteine residues, creating three disulfide bridges that form its secondary beta-pleated sheet structure (Selsted et al., 1985; Ganz, 2003; Lehrer et al., 2005). The three subgroups of defensins are α-, β-, and θ-defensins, categorized by their disulfide bridging array (Fruitwala et al., 2019). Four of the six α-defensins that have been identified are produced by neutrophils, thus they have been denoted human neutrophil peptides (HNP), while α-defensins 5 and 6 are predominantly found in Paneth cells of the small intestine in a subset of species (Jones and Bevins, 1992; Fruitwala et al., 2019). While there have been 34 human β-defensins (hBD) identified using gene analysis, only a small subset of the peptides has been well-characterized (Fruitwala et al., 2019). Human β-defensins 1–3 are secreted by epithelial cells and mononuclear cells while hBD4-6 are designated to specific tissues (Fruitwala et al., 2019). The β-defensins are considered the oldest evolutionary subcategory of the defensins as they can be found in a wide range of species from horseshoe crabs to mammals (Cheng et al., 2014). The α-defensins are thought to have evolved from the β-defensins and have been described in many mammals such as humans, primates, horses, rats, mice, rabbits, and guinea pigs (Wang, 2014). Lastly, the θ-defensins are a unique subcategory of defensins with respect to the synthesis of the peptide. The θ-defensins are translated as two separate peptides that fuse into a circular peptide and are only found in non-human primates (Tang et al., 1999; Cheng et al., 2014).

Of the defensins discovered, it is α-defensins and hBDs that have been shown to play key roles in ocular surface immunity. The α-defensins 1–4 are secreted into the tear film *via* passing and resident neutrophils along the ocular surface (Haynes et al., 1999). Alternatively, hBD 1–3 can be consistently detected in corneal and conjunctival epithelium, and hBD2 and 3 have been found in measurable amounts in human tears (Haynes et al., 1999; McIntosh et al., 2005; Garreis et al., 2010). Additionally, hBD1 can be found in the lacrimal gland and intraocular tissues (Haynes et al., 2000). Human β-defensin 1 is constitutively expressed while hBD2 and 3 are inducible by pathogenic and inflammatory stimuli (McDermott et al., 2003). To highlight their role in ocular surface immunity, an *in vitro* study on human corneal epithelial multilayers demonstrated reduced susceptibility to *P. aeruginosa* penetration following pre-exposure to bacterial antigens, known stimulants of AMPs (Augustin et al., 2011). The same study demonstrated a 3-fold increase in *P. aeruginosa* transversal across multilayered human corneal epithelia following hBD1-3 knockdown (Augustin et al., 2011). Similar *in vivo* effects were seen in corneal epithelia of murine BD3 deficient mice, the ortholog of hBD2, with increased susceptibility and adherence by *P. aeruginosa* when compared to wild-type mice (Augustin et al., 2011). Interestingly, not all hBDs expressed by the ocular surface appear to have a clear antimicrobial effect. Human β-defensin 9 is expressed by ocular surface epithelium, with particularly high concentration in conjunctival epithelium (Mohammed et al., 2010). However, hBD9 has been shown have a different expression pattern entirely and is often downregulated during infection (Otri et al., 2012; Dua et al., 2014).

### Histatins

Histatins are cationic peptides that were first discovered in 1988 when Oppenheim et al. (1988) isolated histatins 1, 3, and 5 from human parotid salivary glands (Oppenheim et al., 1988; Khurshid et al., 2017). Histatin 1 and 3 are encoded on genes HTN1 and HTN3, while histatin 5 is a proteolytic product derived from histatin 3 (Oppenheim et al., 1988). Histatins, named for their histidine-rich composition, are well known for their wound healing effects in saliva, and have similar wound healing effects on the ocular surface, particularly histatin-1 and -5 (Oudhoff et al., 2008; Shah et al., 2017, 2020). Histatin-1 is expressed by both the main and accessory lacrimal glands, and can be detected in human tears, with lower levels of expression found in patients with aqueous deficient dry eye disease (Shah et al., 2016; Kalmodia et al., 2019). When applied to human corneal epithelial cells (HCEC) *in vitro*, histatin-1 showed increased cell surface area and faster wound closure, indicative of enhanced corneal epithelial spreading with minimal toxicity (Shah et al., 2017). Similar results of enhanced wound healing have been replicated in a rabbit model (Oydanich et al., 2018).

### Thymosin

Thymosins contain three subgroups of families denoted as α, β, and γ polypeptides, that were first extracted from the thymus gland of the calf (Goldstein, 2007; Sosne et al., 2016). Thymosin β-4, -10, and -15 are found in humans, with thymosin β-4 being the most abundant and active polypeptide with expression in all tissues except red blood cells (Sosne et al., 2016). Thymosin β-4 was identified as a 43 amino acid G-actin-sequestering peptide with wound healing and anti-inflammatory properties (Goldstein and Badamchian, 2004; Sosne et al., 2016). After detection of thymosin β-4 in HCEC and conjunctival epithelial cells, it was hypothesized that thymosin β-4 could play a role in ocular surface health (Huang et al., 2007a). In an *in vivo* murine model, thymosin β-4 treated corneas had decreased expression of matrix metalloproteinase levels and leukocyte infiltration correlating to decreased inflammation and enhanced corneal wound repair following alkali injury (Sosne et al., 2005). Another study found that thymosin β-4 treated HCEC had increased ability to scavenge reactive oxygen species when compared to controls (Ho et al., 2008). To elucidate an activation pathway responsible for its anti-inflammatory properties, researchers compared thymosin β-4 treated to untreated HCEC *in vitro* following stimulation by TNF-α, a pro-inflammatory cytokine (Sosne et al., 2007). Results demonstrated that thymosin β-4 treated cells inhibited NF-κB phosphorylation and translocation, a commonly utilized pro-inflammatory cascade (Sosne et al., 2004). These anti-inflammatory properties of thymosin β-4 would be of particular benefit for the treatment of ocular surface disease. Treatment with thymosin β-4 significantly reduced signs of dryness in a murine adverse environmental model of dry eye, so appreciably that its effects are now being tested in phase 2 randomized trials (Sosne et al., 2015a,b). It also had significant healing effects and reduced ocular irritation for patients with refractory neurotrophic corneal ulcers (Dunn et al., 2010).

### Psoriasin

Psoriasin (S100A7) is a subset of the S100 proteins family of Ca^2+^ binding proteins, encoded by genes of the epidermal differentiation complex (EDC) located on chromosome 1 (1q21; Mischke et al., 1996; Lesniak and Graczyk-Jarzynka, 2015). Its structure contains two EF-hands, or Ca^2+^ binding motifs, with a high affinity C-terminal containing a 12 amino acid canonical Ca^2+^ binding loop and a 14 amino acid low affinity N-terminal (Lesniak and Graczyk-Jarzynka, 2015). Given its name, psoriasin was first discovered in psoriatic keratinocytes and it is markedly overexpressed in psoriatic tissues compared to normal epidermis (Madsen et al., 1991; Broome et al., 2003; Martinsson et al., 2005). In normal epithelium, it is expressed by superficial, differentiated keratinocytes rather than the basal cells, suggesting its involvement in keratinocyte differentiation (Martinsson et al., 2005). Psoriasin has *in vitro* and *in vivo Escherichia coli*-killing properties in human keratinocytes that is mediated by Zn^2+^ sequestration (Glaser et al., 2005). The toll-like receptor (TLR)-5 ligand, flagellin, was identified as the essential bacterial component needed to induce psoriasin expression in epidermal keratinocytes (Abtin et al., 2008). Psoriasin expression was also induced during epidermal barrier disruption and by cytokines IL-17, IL-22, and TNF-α in *in vitro* and *in vivo* atopic dermatitis models (Glaser et al., 2009a). Besides its antimicrobial activity, psoriasin can influence innate immunity by regulating neutrophil function to produce cytokines and chemokines *via* phosphorylation of mitogen-activated protein kinase (MAPK) p38 and extracellular signal-regulated kinase (ERK; Zheng et al., 2008). In regard to the ocular surface, psoriasin was constitutively expressed in cornea, conjunctiva, nasolacrimal ducts, and lacrimal gland (Garreis et al., 2011). Psoriasin also demonstrated strong staining in meibomian glands on immunohistochemistry (Garreis et al., 2011). Interestingly, the same study detected induction of psoriasin in HCEC following stimulation with *Staphylococcus aureus* and *Haemophilus influenzae* supernatants, but not following stimulation by *E. coli* supernatants (Garreis et al., 2011). Both IL-1β and, to a lesser extent, TNF-α were able to upregulate psoriasin gene expression in HCEC (Garreis et al., 2011).

### Ribonuclease-7

Ribonucealse-7 (RNase-7) is a part of the Ribonuclease A Superfamily, a group of peptides originally isolated from the bovine pancreas (Becknell and Spencer, 2016). RNase-7 is the most potent of the antimicrobial ribonucleases identified and, in addition to its ribonucleolytic activity, also has both angiogenic and immunomodulatory properties (Becknell and Spencer, 2016). RNase-7 has demonstrated antimicrobial activity against vancomycin-resistant *Enteroccocus facium* and can control cutaneous growth of *P. aeruginosa* in an *in vitro* skin keratinocyte model (Harder and Schroder, 2002; Rademacher et al., 2017). RNase-7 also has *in vitro* antibacterial activity against *E. coli*, *P. aeruginosa*, and *S. aureus*, and is upregulated with inflammation caused by UV-B radiation and in the context of atopic dermatitis (Zhang et al., 2003; Gambichler et al., 2008; Glaser et al., 2009b). RNase-7 expression has since been detected in human corneal epithelial cells and upregulation of RNase-7 appears to be mediated through the TAK-1/MAPK pathway following stimulation by the inflammatory cytokine IL-1β (Mohammed et al., 2011).

## Functional Properties of Antimicrobial Peptides

There are myriad functions ascribed to AMPs. This review paper will focus on the functional properties of AMPs that are relevant to microbial keratitis. However, for the functional properties that are less relevant to ocular surface health, such as angiogenesis and apoptosis, please see the following reviews for their current state in the field (Chen et al., 2018; Jafari et al., 2022).

### Antibacterial

**Figure 2:**
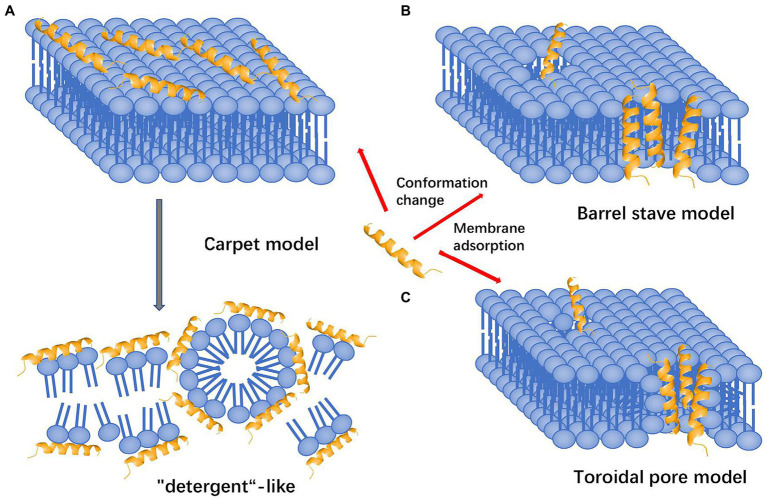
Mechanisms of action attributed to AMPs that lead to disruption of the cell membrane and subsequent death. **(A)** Carpet model: AMPs are attracted to the surface and disrupt the membrane in a detergent-like fashion, destabilizing the phospholipid packaging. **(B)** Barrel stave model: AMPs are attracted to the surface and inserted into the membrane, forming transmembrane helical channels resulting in killing *via* expulsion of water and intracellular contents. **(C)** Toroidal pore model: AMPs are attracted to the surface, embed into the cell membrane and bend to form a ring pore resulting in efflux of ions and small molecules. All models result in death of cell *via* different mechanisms (adapted from Huan et al., 2020).

There is a general consensus that AMPs exert their direct antimicrobial activity through disruption of the pathogen cellular membrane, resulting in cellular content leakage (Matsuzaki, 2019). However, there are multiple proposed theories to explain how this mechanism leads to cell death. These theories include the carpet model, the barrel stave model, and the more widely accepted toroidal pore model (Matsuzaki, 2019). The initial interaction of the AMPs is similar in all three proposed models, whereby AMPs are both electrostatically and hydrophobically attracted to bacterial cell membranes (Matsuzaki et al., 1995). After the initial attraction, the mechanistic theories for membrane disruption diverge. The carpet model describes AMPs as coating the outside surface of the cellular membrane, where they induce negative membrane curvature and charge neutralizing conditions increasing membrane strain, allowing additional AMPs to congregate, resulting in membrane collapse (Figure 2A; Silvestro et al., 1997; Matsuzaki, 2019). By contrast, both the barrel stave model and toroidal pore models focus on insertion of AMPs into the membrane to induce pore formation as the mechanism of action for membrane leakage. However, in the barrel stave model, the walls of the pore are made of purely AMPs, creating a channel for intracellular contents to leak out (Figure 2B; Baumann and Mueller, 1974). This is slightly different from the more widely accepted toroidal pore model, in which the walls of the pore are lined by both polar faces of AMPs and the lipid headgroups of the membrane (Figure 2C; Matsuzaki, 2019). This results in the transient existence of a torus-type pore, allowing an efflux of intracellular contents due to the positive pressure exerted on membrane curvature by the AMPs (Matsuzaki et al., 1998). In general, the cationic nature of the AMPs is beneficial to host immune defenses since it dictates their heavy attraction to the negatively charged bacterial cell membranes that contain acidic phospholipids and abundant lipopolysaccharide (LPS), compared to the zwitterionic phospholipids of host cell membranes (Matsuzaki et al., 1995). In addition, bacterial membranes lack sterols, making them much less resistant to the pore forming effects of AMPs when compared to the cholesterol-rich membrane of host cells (Matsuzaki et al., 1995).

Although the mechanism for membrane disruption primarily involves forced cell lysis and intracellular leakage, these models may not reflect the entirety of AMP mediated microbe killing. Recent studies suggest AMPs can also cause membrane permeabilization by direct inhibition of cell wall biosynthesis and activation of enzymes to lyse cell walls (Bierbaum and Sahl, 1987; Wiedemann et al., 2001; Sass et al., 2010). For example, hBD3 was shown to induce lysis similar to that of the antibiotic vancomycin through an experiment identifying AMP binding to the bacterial membrane precursor in *Staphylococcus* sp., lipid II (Sass et al., 2010). Human β-defensin 3 also induced morphological changes in *S. aureus* similar to treatment with penicillin, whose mechanism of action is known to inhibit cross-linking of peptidoglycans during cell wall synthesis (Harder et al., 2001). Additionally, through ionic interaction, the cationic AMPs can also cause membrane disruption by associating with inhibitors of autolytic enzymes, and therefore activate cell wall lysis (Bierbaum and Sahl, 1987). However, killing kinetics by AMPs seems to be largely dependent on individual microbial strains, peptide concentration and structure, and physiological conditions (Sahl et al., 2005).

### Antiviral

Antimicrobial peptides have been shown to prevent and treat viral infections, however their antiviral activity is not as clearly defined as their bactericidal activity (Park et al., 2018). Rather than simply disrupting membrane integrity, AMPs prevent viral infection at multiple levels including: reduction of infectivity, direct viral inactivation, capsid proteins binding, and inhibition of viral attachment, entry, and replication (Park et al., 2018). This is not an exhaustive list as their antiviral activity appears to be dependent on virus type and physiological conditions (Park et al., 2018).

Human cathelicidin LL-37 has demonstrated protection against human immunodeficiency virus (HIV), influenza A virus (IAV), herpes simplex virus (HSV), rhinovirus (HRV), vaccinia virus (VACV), hepatitis C virus (HCV), respiratory syncytial virus (RSV), zika virus (ZIKV), dengue virus (DENV), and hepatitis C virus (HCV; Ahmed et al., 2019). For the majority of these viruses, LL-37 mediates its attack through the carpet model, in which the viral envelope is dissolved when threshold concentrations of LL-37 are reached (Dean et al., 2010). In a study focused on the antiviral activity of LL-37 against HSV, researchers saw that expression levels of TLR3 and LL-37 were higher in herpetic vesicles in the skin compared to normal controls (Takiguchi et al., 2014). With an understanding of direct viral killing by LL-37, the researchers treated HSV infected human keratinocytes with LL-37 and poly (I:C), a synthetic analog of viral double stranded RNA and a TLR3 ligand (Howell et al., 2004; Takiguchi et al., 2014). Results demonstrated that keratinocytes co-treated with LL-37 and poly (I:C) significantly reduced viral plaque assays *via* TLR3 signaling (Takiguchi et al., 2014).

Like cathelicidins, defensins have also been shown to inhibit HIV, IAV, RSV, and HSV. Additionally, defensins have been found to impede human adenovirus (HAdV), papilloma virus (HPV), and severe acute respiratory syndrome coronavirus (SARSC; Ahmed et al., 2019). Defensins have a wide repertoire of antiviral activity in which they directly or indirectly intervene in the viral life cycle. Some of the major studies of AMP antiviral activity of the ocular surface investigated the effect of defensins against HSV, a double-stranded DNA virus known to cause epithelial keratitis of the cornea (Koujah et al., 2019). Additionally, all human defensins (with the exception of hBD1 and 2) significantly inhibit HSV-2 infection in human cervical epithelial cells at 25 and 50 μg/ml (Hazrati et al., 2006). The methods of viral inhibition include prevention of viral binding, entry, and post-entry events (Hazrati et al., 2006). Specifically, by binding the cell surface receptors glycoprotein D or heparin sulfate, the α-defensins and hBD3 were able to inhibit infection by HSV (Hazrati et al., 2006). Especially intriguing were the post-entry effects seen as HSV infected cells expressed less viral fusion protein when treated with human alpha defensins [human neutrophilic peptide 1 (HNP-1) or human alpha defensin 5 (HD5)], compared to treatment with a defensin-free buffer (Hazrati et al., 2006). Studies such as these show the exciting potential for AMPs to be effective microbicides against pervasive viruses known to commonly infect the eye, including HSV.

### Antifungal

AMPs have demonstrated antifungal activity as well with action against *Fusarium*, *Aspergillus*, *Cryptococcus*, and *Candida*, which are increasingly resistant fungal pathogens of the eye. In a murine model of *Fusarium* keratitis, researchers observed increased expression of murine β-defensins and cathelin-related antimicrobial peptide (CRAMP) at the onset of infection and faster recovery rates when compared to knockdown or knockout mice that experienced significantly augmented disease severity (Kolar et al., 2013). Another study concluded that defensins have also been shown to significantly damage *Cryptococcus* species. After treatment with various AMPs, the β-defensins proved to be among the most effective AMPs at slowing metabolic activity and decreasing fungal mass with planktonic cryptococci being more susceptible to damage than cryptococcal biofilms (Martinez and Casadevall, 2006). The mechanism for how AMPs interfere with fungal invasion has yet to be fully understood, but researchers have observed cell membrane disruption and interference with fungal adhesion (Tsai et al., 2011; Goncalves et al., 2017). For example, human cathelicidin (LL-37) has been shown to target *C. albicans* by binding mannan, the carbohydrate component of yeast cell walls, inhibiting fungal adhesion and aggregation in both *in vitro* and *in vivo* models (Tsai et al., 2011). Current treatment for fungal keratitis includes topical natamycin, amphotericin, or, for deep penetrating hyphae, voriconazole (Joseph and Sharma, 2016). However, with current increases in resistance to antifungal medications, researchers are pursuing the use of AMPs as an attractive alternative in patients undergoing corneal transplantation (Sara et al., 2016).

### Chemotaxis

Chemoattraction is the movement of a cell in response to a chemical gradient in which the cell moves toward a high concentration of ligands. AMPs have demonstrated chemotactic properties for various cells of the innate and adaptive immune system. Based on the observation that interleukin-8 (IL-8) induced neutrophilic degranulation followed by T-cell infiltration, a 1996 study attempted to identify the neutrophil-derived chemoattractant factors, postulating that these were the cause for T-cell migration. The chemoattractant factors identified were hBD1-3, with the first two defensins having potent chemotactic capabilities for human T cells (Chertov et al., 1996). More specifically, mouse beta-defensin 14 (mBD14), the orthologue of hBD3, has been shown to be chemotactic for cells expressing mouse CC-chemokine receptor 6 (CCR6; Rohrl et al., 2008). The same chemoattractant effect was seen for hBD2, which utilized CCR6 to recruit human neutrophils that were pretreated with TNF-α (Niyonsaba et al., 2004).

After the discovery of defensin-mediated chemoattraction, researchers hypothesized that LL-37 would also have potent chemotactic activity. Indeed, LL-37 promoted the chemotaxis of various leukocytes, through Ca^2+^ mobilization in human monocytes, as well as the recruitment of neutrophils and T-cells *via* formyl peptide receptor-like 1 (FPRL1; Yang et al., 2000). Additionally, pulmonary researchers demonstrated that LL-37 attracts human peripheral blood eosinophils and neutrophils, cell types that characterize respiratory diseases such as asthma or chronic obstructive pulmonary disease, *via* FPRL1 (Tjabringa et al., 2006). This finding was confirmed when FPR antagonists inhibited LL-37-mediated chemotaxis (Tjabringa et al., 2006). In regard to the ocular surface, LL-37 has been shown to promote HCEC chemotaxis *via* the protein kinase pathways (PKC; Griffith et al., 2013). The ability of AMPs to chemoattract cells of the adaptive immune system creates a more streamlined inflammatory response between the innate and adaptive immunity, however, more studies are needed to further investigate the role of AMP-mediated chemotaxis on the protection of the ocular surface.

### Wound Healing

Of the AMPs present on the ocular surface, thymosin β-4 and histatins have been promoted corneal wound healing. For re-epithelialization to occur, there must be epithelial cell migration and proliferation. In an *in vitro* study on human conjunctival epithelial cells, thymosin β-4 stimulated cell migration in a dose-dependent manner when compared to controls (Sosne et al., 2002). Thymosin β-4 treated cells also expressed increased laminin-5 deposition, an extracellular protein that initiates hemidesmosome formation and basement membrane attachment (Sosne et al., 2002). Similar benefits were demonstrated in the *in vivo* murine model following alkali burns. Thymosin β-4 treated mice had improved corneal clarity and decreased matrix metalloproteinases and infiltrating leukocytes, thereby promoting corneal wound repair following injury (Sosne et al., 2005). Researchers discovered that thymosin β-4 utilizes the ERK1/2 signaling pathway *via* a P2X7 receptor-mediated calcium influx to enhance HCEC proliferation and migration (Yang et al., 2020). Besides thymosin β-4, the histatin family also has potent wound healing capabilities. *In vitro* HCECs treated with histatin-1 demonstrated increased corneal epithelial spreading and pathfinding during re-epithelialization of a scratch wound (Shah et al., 2017). Histatin-5 had similar *in vitro* properties, demonstrating enhanced cellular migration of HCEC in a sprouting assay and faster rate of scratch closure in human corneal limbal epithelium (Shah et al., 2020). Histatin-5 also promotes cellular spreading, exhibited by an increase in surface area of treated HCECs (Shah et al., 2020). In the same *in vitro* study, histatin-5 was determined to utilize the phosphorylated ERK1/2 pathway (Shah et al., 2020). Histatins have similar efficacy in the *in vivo* rabbit corneal injury model. Following corneal injury with ethyl alcohol, rabbits treated with varying concentrations of histatin-1 demonstrated a faster recovery rate and a higher percent recovered area when compared with controls (Oydanich et al., 2018). Besides thymosin β-4 and histatins, LL-37 and hBD2 and 3 also play a role in wound healing. *In vivo* skin wounds demonstrated high levels of LL-37 in both inflammatory cells and migrating epithelium, and treatment with LL-37 induced cell proliferation and migration in airway epithelial cells (Heilborn et al., 2003b; Shaykhiev et al., 2005). In a study on corneal epithelialization in the context of diabetes, high-glucose attenuated wounds had enhanced LL-37 expression, which in response, prolonged the effects of epidermal growth factor receptor (EGFR) signaling and its ability to hasten scratch wound closure (Yin and Yu, 2010). Interestingly, low levels of LL-37 are present in chronic corneal ulcers, indicating that re-epithelialization may be partially dependent on LL-37 concentration (Heilborn et al., 2003a). Human β-defensin 2 has shown to augment wound healing in keratinocytes and hBD3 can stimulate intestinal epithelial migration *in vitro* (Otte et al., 2008; Hirsch et al., 2009). Additionally, hBD2 has shown to be upregulated in the re-epithelialized cornea, leaving researchers to hypothesize that it plays a key role in wound healing (McDermott et al., 2001). The ability of AMPs to function as immunomodulators and accelerate wound healing is fundamental to their role as modulators of the host immune system and can be leveraged in the future for therapeutic use.

### Antibiofilm

Biofilms are aggregations of microbes that secrete extracellular polymeric substances (EPS) that allow for the adhesion of carbohydrates, proteins, lipids, and nucleic acids (Khatoon et al., 2018). This mixture of nutrients serves as a platform for bacteria to engage with, resulting in the upregulation of intracellular signaling and proliferation (Khatoon et al., 2018). Biofilms present a foreboding clinical problem with their growing antibiotic resistance and their tendency to accumulate on medical devices, such as contact lenses (Khatoon et al., 2018). Researchers have observed that biofilm formation of *S. epidermidis* was inhibited by human cathelicidin and that bacterial attachment to artificial surfaces was diminished at concentrations of LL-37 similar to human plasma levels (Hell et al., 2010). Using microarray technology, it was revealed that the mechanism by which LL-37 inhibits bacterial strains, like *P. aeruginosa*, involves the down-regulation of genes responsible for quorum sensing which are essential for biofilm formation (Overhage et al., 2008). The ability of AMPs to disrupt biofilm formation will become increasingly relevant as more therapeutic interventions for the ocular surface will rely on drug delivery devices on which bacteria could form biofilms.

## Regulation of Antimicrobial Peptide Expression

AMPs are considered frontline defenses of the innate immune system, as they are constitutively expressed by epithelial cells of the ocular surface, intestine, skin, respiratory, and the reproductive systems to kill pathogens directly (Gallo and Hooper, 2012). However, many pathogenic stimuli, inflammatory mediators, and some small molecules can initiate a signaling cascade resulting in the upregulation of endogenous AMP expression. Given this knowledge, the ability to modulate AMP expression could serve as a therapeutic strategy to mitigate the pathologic consequences of infection. The following sections will include studies from multiple organ systems, including the ocular surface, to provide a more complete overview of how AMP gene expression is regulated and how modulation of their expression could be employed for therapeutic use.

### Pathogenic Stimulation

**Figure 3:**
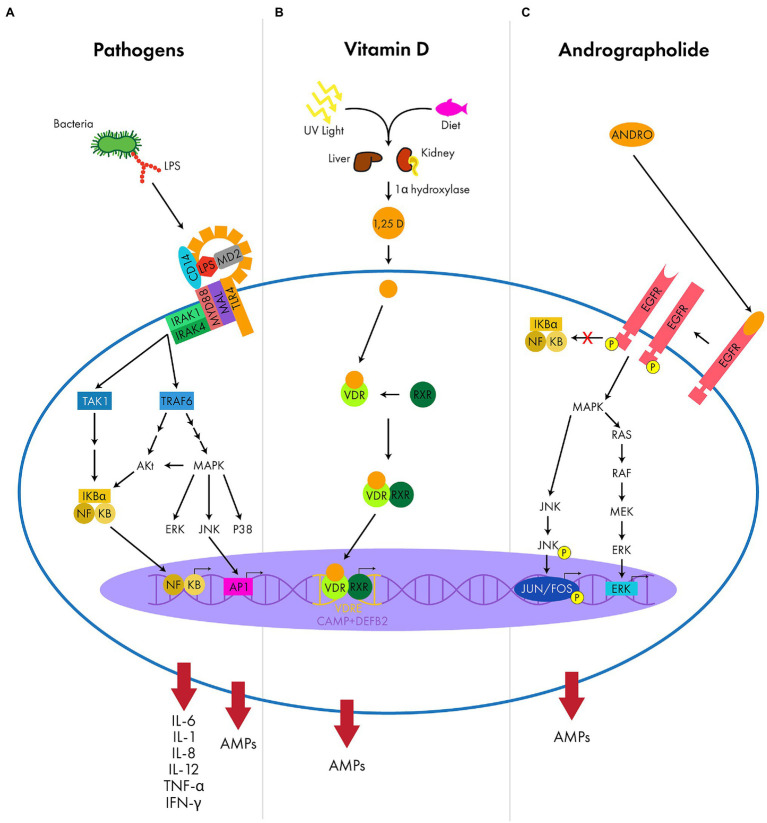
Examples of the signaling molecules and pathways that result in upregulation of AMP expression. **(A)** The LPS complex (TLR4, CD14, and MD2) recognizes PAMPS (LPS) and uses adapter proteins (MyD88 and MAL) to activate intracellular protein kinases (IRAK1 and IRAK4). These intracellular kinases trigger a signaling cascade (TAK1, TRAF6, Akt, MAPK and the subfamilies ERK/JNK/P38) initiating transcription factors (NF-κB and AP1) to upregulate AMPs and pro-inflammatory cytokines (IL-6, IL-1, IL-8, IL-12, TNF-α, and IFN-γ). **(B)** Vitamin D is absorbed from the conversion of UV light in the skin or from the diet and is metabolized by various enzymes (1α-hydroxylase) of the kidney and liver to produce the active metabolite, calcitriol (1,25 D). Calcitriol will bind intracellular receptors (VDR and RXR) that heterodimerize and target specific DNA sequences with VDREs, such as *CAMP* and *DEFB2*, to upregulate AMP expression. **(C)** Andrographolide (ANDRO) activates a ligand-induced dimerization of epidermal growth factor receptor (EGFR), allowing for tyrosine kinase activity and auto-phosphorylation. This triggers an intracellular signaling cascade (MAPK and subfamilies RAS/RAF/MEK/ERK and JNK), activating transcription factors (JUN/FOS and ERK) to upregulate AMP expression without the activation of the pro-inflammatory NF-κB pathway.

AMP expression can be induced through the activation of pathogen recognition receptors (PRRs), such as TLRs, which recognize conserved structural pathogen motifs known as pathogen associated molecular patterns (PAMPs; Mogensen, 2009; Gallo and Hooper, 2012). Some TLRs reside at the cell surface (TLR1, -2, -4, -5, -6, -10) and recognize unique bacterial products, such a LPS or flagellin, while others reside in endosomes (TLR3, -7, -8, -9) and recognize exogenous nucleic acids (Mogensen, 2009). Once bound to its endogenous or exogenous ligand, TLRs will induce an intracellular signaling cascade that is classified as either myeloid differentiation primary response protein 88 (MyD88)-dependent or MyD88-independent. MyD88 is an adapter molecule used by TLRs as a co-stimulatory protein that helps shape the cellular response to PAMPs. Depending on the TLR activated, the subsequent signaling cascade involves multiple protein kinases, such as mitogen-activated protein kinase (MAPK). Ultimately, this cascade activates transcription factors, such as nuclear factor kB (NF-κB), activator protein-1 (AP-1), and interferon regulatory factors 3 and/or 7 (IRF3/7), resulting in the coordinated activation of both the host’s innate and adaptive immune response by producing proinflammatory cytokines, type 1 interferons, and AMPs (Figure 3A; Gomariz et al., 2010; Prasad et al., 2019).

Given this activation pathway, there have been multiple studies demonstrating upregulation of AMP expression at the ocular surface and in other organ systems with pathogenic stimulation. Defensins and LL-37 become upregulated in various leukocyte and epithelial systems, such as the skin and intestine, following exposure to pathogens (O’Neil et al., 1999; Rivas-Santiago et al., 2008; Lai et al., 2010; Koon et al., 2011). Similar trends can be seen at the ocular surface. AMPs are consistently upregulated in HCEC in the presence of common bacterial keratitis pathogens such as *Streptococcus pneumoniae*, *Corynebacterium pseudodiphtheriticum*, *P. aeruginosa* (Augustin et al., 2011; Redfern et al., 2011; Evans and Fleiszig, 2013; Roy et al., 2015; Sharma et al., 2019). In HCEC treated with TLR agonists, there was an upregulation of hBD2 and LL-37 expression, with LL-37 having efficacy killing *P. aeruginosa* (Redfern et al., 2011). Similarly, in HCEC infected with *S. pneumonia*, LL-37 expression was induced *via* transcription factor STAT3 activation and had effective killing *in vitro* (Sharma et al., 2019). Induction of LL-37 and hBD1-3 was also seen in limbo-corneal fibroblasts post-infection with mycobacteria (Castañeda-Sánchez et al., 2013). PAMPs from fungal pathogens also induce AMP expression. Human corneal epithelial cells upregulated hBD2 and LL-37 following exposure to heat inactivated *Fusarium solani* (Kolar et al., 2017). This upregulation was determined to be dependent on TLR2 and dectin-1, a PRR, since knockdown of TLR2 and dectin-1 in HCEC abrogated this response. Similarly, heat-killed *Candida albicans* dose-dependently stimulated LL-37, hBD2, and hBD3 production in HCEC (Hua et al., 2014).

Knowing that PAMPs stimulate AMP expression, researchers have turned to methods of topical application of de-activated PAMPs that could pre-treat the cornea to resist infection by microbes. Following topical application of flagellin, the principal protein of bacterial flagella, mice corneas had improved protection from candida keratitis (Gao et al., 2011). Using TLR5, flagellin was able to induce CAMP upregulation, and once CAMP was totally ablated the protective effects against *C. albicans* were abolished (Gao et al., 2011). In parallel, corneal fibroblasts that were pre-treated with LPS expressed elevated levels of thymosin β-4 upon subsequent challenge with *Aspergillus fumigatus* (Xiaoyan et al., 2011). These studies are just a few examples demonstrating the close relationship between activation of TLRs by PAMPs, induction of AMP synthesis, and how their induction can be modulated to augment the innate immune response.

### Inflammatory Stimulation

Similar to the mechanisms used to stimulate AMP secretion *via* TLR activation, inflammatory cytokines and mediators have also been shown to induce AMP expression to modulate host immune defense. When innate immune cells encounter pathogens, a signaling cascade induces secretion of inflammatory cytokines to signal cells of the adaptive immune system (Kolls et al., 2008). These cytokines work not only to modulate the host’s immune response, but also to upregulate AMP expression (Kolls et al., 2008). The interleukin-1 (IL-1) family of cytokines is one of the best-known inducers of AMPs. Evidence of the regulatory pathway responsible for cytokine upregulation of AMPs in multiple organ systems, including the ocular surface, is thought to involve MAPK and NF-κB transcription factors (Krisanaprakornkit et al., 2002; McDermott et al., 2003; Kao et al., 2004). Relevant to the ocular surface, McDermott et al. demonstrated upregulation of hBD2 gene expression in HCEC when treated with IL-1β and TNF-α (McDermott et al., 2003). The upregulation of hBD2 expression in HCEC was abolished in the presence of NF-κB inhibitors and partially blocked with both MAP kinase and JNK inhibitors independently (McDermott et al., 2003). Interestingly, hBD2 is found to have higher expression in inflamed conjunctiva of patients with pterygium, likely due to stimulation by cytokines (Karadag et al., 2017). The importance of IL-1 as an AMP stimulant was highlighted in the murine model of experimental dry eye, where IL-1 receptor deficient mice demonstrated non-existent to decreased levels of mBD2 in cornea and conjunctiva when compared to wild type mice (Narayanan et al., 2008) Interestingly, local application of a newly discovered subfamily of cytokines, IL-36γ, stimulated expression of mBD3 in murine corneas while simultaneously inhibiting IL-1β (Gao et al., 2018). These findings demonstrate the need to further investigate the exact mechanisms of AMP upregulation by cytokines to better define the fundamental role of AMPs in modulating host immune function to better inform therapeutic strategies (Moon et al., 2002; Albanesi et al., 2007).

### Small Molecules

There are many small molecules known to upregulate AMP expression including: vitamin D, fatty acids (ex. butyrate; Raqib et al., 2006; Nakatsuji et al., 2010; Jiang et al., 2013; Sunkara et al., 2014; Xiong et al., 2016; Wang et al., 2018; Schulthess et al., 2019; Takakuwa et al., 2019; Zhang et al., 2020; Schaefer et al., 2022), amino acids, (Sherman et al., 2006; Ren et al., 2016; Zhang et al., 2017; Schaefer et al., 2022) trace nutrients (ex. zinc; Wang et al., 2006; Talukder et al., 2011; Liu et al., 2014), and other less common molecules, like curcumin (Guo et al., 2013; Liu et al., 2020), resveratrol (Ravagnan et al., 2013), isoflavones (Srisomboon et al., 2017), genistein (Park et al., 2014), and andrographolide (Sechet et al., 2018). This section highlights the mechanisms of (1) vitamin D-induced upregulation of cathelicidin and (2) andrographolide upregulation of hBD3. Importantly, upregulation of AMP expression with vitamin D and andrographolide has been shown to occur without the release of inflammatory mediators that would exacerbate ocular surface disease and potentially impair vision.

### Vitamin D

The vitamin D receptor (VDR) pathway proteins are widely expressed among epithelial and immune cells and the VDR pathway plays a critical role in the induction of AMP expression. There is evidence that activation of the VDR pathway directly upregulates AMP expression, specifically, LL-37 and hBD2 (Wang et al., 2004; Gombart et al., 2005). Vitamin D_3_ can either be ingested from the diet or synthesized by the skin with UV light exposure and subsequent conversion to the active form, 1,25 dihydroxyvitamin D(1,25D; Prosser and Jones, 2004). Binding of 1,25D to the cytosolic VDR results in the heterodimerization of the VDR with the retinoid X receptor (White, 2010; Svensson et al., 2016). Then this complex binds to the vitamin D response element (VDRE) located in the regulatory regions of the *CAMP* and *DEFB2* genes, stimulating transcription and upregulation of AMP expression (Figure 3B; Wang et al., 2004; Svensson et al., 2016). Utilizing this pathway, vitamin D is a potent inducer of LL-37 and could serve as a therapeutic target. In fact, LL-37 plasma concentrations directly reflect systemic vitamin D_3_ concentrations with higher values of vitamin D_3_ correlating with higher levels of LL-37 expression (Dixon et al., 2012). Upregulation of LL-37 expression with vitamin D supplementation has been shown to reduce the growth of mycobacteria *in vitro* and aid wound healing in human skin *in vivo* (Martineau et al., 2007; Heilborn et al., 2010). The role of vitamin D in regulating LL-37 expression has also been demonstrated in corneal epithelial cells. Previous studies have applied vitamin D metabolites to corneal epithelial cells *in vitro* and observed the production of the active metabolite, 1,25 vitamin D_3_, which enhanced LL-37 expression while attenuating pro-inflammatory cytokines and metalloproteinases (Reins et al., 2015). Furthermore, topical application of vitamin D in a mouse model of corneal wound healing resulted in delayed wound closure, increased infiltration of neutrophils, and increased expression of CRAMP (Reins et al., 2016). The increased neutrophil infiltration may have been responsible for the delayed re-epithelialization as vitamin D also upregulated the neutrophil chemoattractant CXCL (Reins et al., 2016). Despite the delay in wound healing from the neutrophilic infiltration, the increased expression of CRAMP did exert the beneficial effect of blocking pathogen entry in a non-sterile wound environment, providing protection to the exposed epithelium (Reins et al., 2016). When corneal epithelial cells were infected by *Fusarium solani*, researchers observed an upregulation of the VDR *via* the TLR2/1-VDR pathway, identifying the role of the VDR pathway as a potential target for upregulating CAMP to treat fungal keratitis (Cong et al., 2015). As an inducer of LL-37, vitamin D shows promising potential as a topical therapeutic. However, not all studies have reached similar conclusions about the positive therapeutic effect of vitamin D, thus additional studies into the role of vitamin D on the ocular surface innate immune system is warranted (Sel et al., 2016).

### Andrographolide

Andrographolide is a small molecule extracted from the herb *Andrographis paniculata* that has been used for centuries across some Asian cultures as a remedy to treat respiratory and gastrointestinal bacterial infections (Tan et al., 2017). A recent study identified the inducible effects of andrographolide on hBD3 in colonic epithelial and organoid cell lines (Sechet et al., 2018). There was a dose-dependent increase in mRNA expression of hBD3 when cells were treated with andrographolide and a 100-fold increase in hBD3 peptide expression was identified at 75 and 100 μM of andrographolide (Sechet et al., 2018). Using EGFR inhibitors, they determined that the EGFR-ERK (extracellular-signal-regulated kinase)-JNK (c-Jun N-terminal kinase) pathway was responsible for the upregulation of hBD3 expression. After inducing dimerization of EGFR, andrographolide activated the MAPK intracellular signaling cascade, a pathway responsible for cell proliferation, stress signaling, and AMP expression (Figure 3C; Sechet et al., 2018). Interestingly, andrographolide induced hBD3 expression without activating the pro-inflammatory effects of the NF-κB pathway (Sechet et al., 2018). They also observed that the effects of andrographolide in boosting hBD3 expression could be further increased when synergized with another molecule from natural pharmacopeia, isoliquiritigenin (Sechet et al., 2018). Similarly, other research groups found that andrographolide could also induce hBD2 expression in intestinal and lung epithelial cells (Shao et al., 2012; Xiong et al., 2015). In addition, these groups observed a less pronounced upregulation of hBD2 expression when co-treated with inhibitors of the MAPK pathway, p38 (Shao et al., 2012; Xiong et al., 2015). Moving forward, studies that utilize andrographolide to induce hBDs in corneal epithelial cells would serve as a proof-of-concept for implementing new therapeutics to target resistant infectious keratitis while decreasing the pro-inflammatory mediators that compromise vision.

## Therapeutic Use of Antimicrobial Peptides

### Exogenous Use of Antimicrobial Peptides

Given their great efficacy against pathogens, some researchers have taken the approach of directly applying both natural and synthetic AMPs to wounds (Clemens et al., 2017). Several studies have shown that application of exogenous natural AMPs can have immunomodulatory and protective effects against infectious keratitis (Gao and Yu, 2015; Kolar et al., 2015; Shah et al., 2017). Furthermore, it has been observed that AMPs can synergize with each other and additional antibacterial enzymes (Nagaoka et al., 2000; Singh et al., 2000; Wu et al., 2009; Chen et al., 2019). From this, researchers hypothesized that AMPs could synergize with current antibiotic therapies to potentiate bactericidal effects (Li et al., 2014; Carion et al., 2020). These effects can be seen at the ocular surface when researchers applied thymosin β-4 and ciprofloxacin to HCEC *in vitro* (Carion et al., 2020). While thymosin β-4 did not have direct antibacterial effects, it synergized with ciprofloxacin to upregulate other AMPs and related molecules for improved disease response (Carion et al., 2020). However, utilizing exogenous AMPs can cause cytotoxic effects at high concentrations, which are often necessary to see beneficial effects (Huang et al., 2006). Given this fine balance, some scientists have attempted altering AMP structure or creating synthetic AMPs (Kowalski et al., 2016; Clemens et al., 2017). For example, Brilcidin (PMX30063), a defensin mimetic, demonstrated *in vitro* activity against gram-positive bacteria in ocular isolates collected from patients with keratitis and endophthalmitis (Kowalski et al., 2016). *In vivo*, Brilcidin 0.5% was equally efficacious to vancomycin in reducing methicillin-resistant *S. aureus* (MRSA) in rabbit eyes (Kowalski et al., 2016). Given the success in studies on the exogenous application of AMPs, researchers have experimented with delivering AMPs in different vehicles, including contact lenses, hydrogels, and liposomes (Kumar et al., 2011; Dutta et al., 2016; Gallagher et al., 2016; Kennedy et al., 2020; Kalaiselvan et al., 2021). In an *in vivo* rabbit model, melimine-coated contact lenses significantly reduced *P. aeruginosa* microbial keratitis, had lower ocular scores, and improved ocular signs compared to controls (Dutta et al., 2016). However, researchers observed a corneal staining response seen with melimine-coated contact lenses, and decided to create Mel4, a shorter version of the melimine peptide that has the hydrophobic amino acids removed (Dutta et al., 2018; Willcox et al., 2020). The success of Mel4 coated contact lenses in preclinical testing, led to phase I/II/III clinical trials where the Mel4 contact lenses showed a 50% reduction in corneal infiltrative events, defined as inflammation caused by microbial colonization of contact lenses (Willcox et al., 2020). Studies such as these are promising, however, this therapeutic potential can be highly variable. Additionally, their utilization is limited by the cost of production, bioavailability, cytotoxicity, and pharmacokinetic stability (Mannis, 2002; Huang et al., 2006; Brandt et al., 2007; Dutta and Das, 2016). Until circumventions are made, such as using D amino acids to improve pharmacokinetic stability or creating shorter peptides to improve production costs and decrease cytotoxicity, the use of synthetic peptides may not be the most efficient therapeutic approach (McPhee et al., 2005; Liu et al., 2008). An alternative approach focused on the modulation of endogenous AMP expression may serve as a viable treatment option for patients with infectious disease, particularly through the topical application of AMP induction molecules at the ocular surface to upregulate the innate immune system.

### Modulation of Endogenous Antimicrobial Peptide Expression

Through our understanding of AMP regulation pathways, there is a potential to modulate endogenous AMP expression with small molecules as either a prophylactic measure to prevent infection or as a method to treat infection. Outside of the use of narrowband ultraviolet B treatment to increase systemic vitamin D levels (Vahavihu et al., 2010) or the direct supplementation of vitamin D, (Peric et al., 2009) there is a paucity of studies focused on modulating AMP expression *in vivo*. This represents an unexplored area for developing a novel therapeutic approach for the treatment of microbial keratitis. Additionally, the antimicrobial activity of AMPs have been shown to be potentiated when combined with traditional antibiotic therapy (Darveau et al., 1991). More specifically, co-treatment of hBD3 with either amoxicillin, chlorhexidine or metronidazole resulted in synergistic antimicrobial activity against *Streptococcus mutans*, *Actinobacillus actinomycetemcomitans*, and *Porphyromonas gingivalis* compared with single agent treatment alone (Maisetta et al., 2003).

## Conclusion

Antimicrobial peptides are critical to maintaining the health of the ocular surface and prevent infection. This review highlights the key AMPs expressed by the corneal and conjunctival epithelium in health, their method of action and regulation of expression. Future studies are warranted to evaluate the therapeutic benefit of exogenous AMP delivery as well as the modulation of endogenous peptide expression. The eye represents an ideal organ system for these therapeutic approaches due to the direct delivery of compounds to the site of infection and the cell types required to halt progression. Researchers should continue to explore the intricacies of AMP signaling pathways and regulation in addition to providing vehicles for extended drug delivery such as nano-particle based vectors, liposomes, and drug-eluting contact lenses (Lakhani et al., 2018; Xu et al., 2018; Bhattacharjee et al., 2019; Martin-Serrano et al., 2019). In doing this, ocular pharmacology could shepherd in a new therapeutic approach for the treatment of microbial keratitis while simultaneously limiting the potential for antimicrobial resistance.

## Author Contributions

AS and BL: conceptualization and writing (original draft preparation). All authors contributed to the article and approved the submitted version.

## Funding

This work was supported by the Summer Training in Advanced Research from University of California, Davis, School of Veterinary Medicine (AS) and the National Institutes of Health K08EY028199 (BL) and R01EY019970 (CM and ST).

## Conflict of Interest

The authors declare that the research was conducted in the absence of any commercial or financial relationships that could be construed as a potential conflict of interest.

## Publisher’s Note

All claims expressed in this article are solely those of the authors and do not necessarily represent those of their affiliated organizations, or those of the publisher, the editors and the reviewers. Any product that may be evaluated in this article, or claim that may be made by its manufacturer, is not guaranteed or endorsed by the publisher.
